# Using urine FTIR spectra to screen autism spectrum disorder

**DOI:** 10.1038/s41598-023-46507-z

**Published:** 2023-11-09

**Authors:** Neslihan Sarigul, Leyla Bozatli, Ilhan Kurultak, Filiz Korkmaz

**Affiliations:** 1 Institute of Nuclear Science, Hacettepe University, Ankara, Turkey; 2 Department of Child and Adolescent Psychiatry, Faculty of Medicine, Trakya University, Edirne, Turkey; 3 Department of Nephrology, Faculty of Medicine, Trakya University, Edirne, Turkey; 4 Biophysics Laboratory, Faculty of Engineering, Atilim University, Ankara, Turkey

**Keywords:** Biological physics, Autism spectrum disorders

## Abstract

Autism spectrum disorder (ASD) is a heterogeneous neurodevelopmental disorder caused by multiple factors, lacking clear biomarkers. Diagnosing ASD still relies on behavioural and developmental signs and usually requires lengthy observation periods, all of which are demanding for both clinicians and parents. Although many studies have revealed valuable knowledge in this field, no clearly defined, practical, and widely acceptable diagnostic tool exists. In this study, 26 children with ASD (ASD+), aged 3–5 years, and 26 sex and age-matched controls are studied to investigate the diagnostic potential of the Attenuated Total Reflectance-Fourier Transform Infrared (ATR-FTIR) spectroscopy. The urine FTIR spectrum results show a downward trend in the 3000–2600/cm region for ASD+ children when compared to the typically developing (TD) children of the same age. The average area of this region is 25% less in ASD+ level 3 children, 29% less in ASD+ level 2 children, and 16% less in ASD+ level 1 children compared to that of the TD children. Principal component analysis was applied to the two groups using the entire spectrum window and five peaks were identified for further analysis. The correlation between the peaks and natural urine components is validated by artificial urine solutions. Less-than-normal levels of uric acid, phosphate groups, and ammonium (${NH}_{4}^{+}$) can be listed as probable causes. This study shows that ATR-FTIR can serve as a practical and non-invasive method to screen ASD using the high-frequency region of the urine spectrum.

## Introduction

Autism spectrum disorder (ASD) is a heterogeneous neurodevelopmental disorder characterized by impaired communication and social interaction, as well as repetitive and unusual sensory-motor behaviours^1^. In the United States, in 2018, its prevalence among children aged eight years increased to 1 in 44, and ASD is 4.2 times more common among boys than among girls^2^. Multiple factors have been proposed to contribute to the development of ASD, such as biological, neurological, metabolomic, genetic^3^, immunity, prenatal, and environmental exposures, but the pathophysiology of autism is still unknown^4^. Despite revolutionary developments in biotechnology during the last several decades, ASD is still diagnosed through observation, parental interviews, and screening tools. This diagnosis process is usually time-consuming, challenging, and tiresome for clinicians, children, and parents^5,6^. In addition, there is no curative treatment for ASD. Early identification of autistic children and starting supportive therapy as early as possible are the most crucial factors for better outcomes^7–10^. Instead, the biomarker-based identification of ASD could make early diagnosis possible and allow for timely intervention. However, no clearly defined diagnostic tool is available for this purpose yet. For this reason, ASD has been extensively researched in different fields, such as genetic sequencing and neuroimaging, as well as proteomic, immune system, and metabolomic studies^11,12^. Phenylactic acid^13^, benzoyl phosphate^13,14^, 3-oxoglutaric acid^13^, carboxylic acid^15^, tryptophan^6,16^, purine^6^, kynurenine^6,16^, taurine^17,18^, N-methyl nicotinamide^19^, p-cresol^6^, Hippurate^19,20^, glutamate^19^, indole acetic metabolites^21^, methionine cycle ^22^, glutathione metabolism^23,24^, lipid metabolism^25^, homocysteine metabolism^25^, and urea cycle^22,25^ have all been investigated in blood and urine as a possible biomarker for ASD. Since these substances are not common to all children diagnosed with ASD, accurate diagnosis is still a challenging issue that needs further research.

Obtaining urine and blood samples differ owing to certain features, such as being non-invasive, sterile, and easily available^26^. As for urine, it can provide potentially important metabolic information^26^. Recent studies have focused on the potential of urinary metabolomics in identifying/separating the subgroups of ASD^6,17,20,27–30^. Different methods such as mass spectroscopy (MS), MS-TOF, nuclear magnetic resonance (NMR), and chromatography combined with MS (GC–MS, LC–MS/MS) have been used for this purpose^17,20^. These expensive and sophisticated techniques have been successfully used to quantify a particular substance, but they are too complex to be used on a daily basis by hospital staff.

Another solution, vibrational spectroscopy, is a fast, label-free, and practical method providing molecular information about the sample structure. With the advances in vibrational spectroscopy, biofluids such as urine can be analyzed rapidly to identify disease-related abnormalities. Attenuated Total Reflectance-Fourier Transform Infrared spectroscopy (ATR-FTIR), a vibrational spectroscopy method, has been successfully applied for the analysis of urine^31,32^. Its use in the quantification of urinary urea, protein, creatinine, and cysteine^33,34^, and in cancer investigations using urine samples^35–37^ has been shown previously.

In the available literature, studies mostly involve detailed component-by-component analysis of urine rather than looking from a broader aspect. It is usually aimed at finding a new biomarker or to comparing the number of suspicious metabolites with control groups. However, looking from a broader aspect rather than just one piece of the puzzle can provide an advantage in reaching correct diagnoses, especially in disorders such as ASD, which is associated with alterations in many substances within the urine. In this study, the diagnostic potential of ATR-FTIR spectroscopy is tested using the urine samples obtained from ASD+ children (aged 3–5 years) and healthy, typically developing (TD) children. In the literature, data on children with ASD in this age range is quite limited. A multianalyte analysis approach is preferred for the comparison of the spectra.

## Materials and methods

### Sample collection

This study was approved by the Atilim University Human Studies Ethics Committee (Ref. Number: 59394181-604.01.01-8509). All experiments were performed in accordance with relevant guidelines and regulations. After the participants’ parents signed an informed consent form, first-morning midstream urine samples were collected from 26 Turkish children aged 3–5 years newly diagnosed with ASD, as well as from 26 sex-, BMI-, and age-matched controls showing typical development after at least an 8 h fasting period. The parents answered a list of questions about their children regarding their general eating habits, food their children are obsessed with, and the use of any prescribed medication. Children who did not use any medications, have a healthy diet with no particular food obsessions, and who had toilet training were included in the study. Firstly, the urine samples were collected in clean, dry specimen tubes according to the urine collection guidelines. Then, a dipstick test (Mission Acon, San Diego, California) was used to screen the urinary parameters. Each urine sample was aliquoted into 2 mL tubes and stored at – 20 °C until measured with ATR-FTIR. The BMI was calculated by weight/height^2^ for all participants.

The Diagnostic and Statistical Manual of Mental Disorders, Fifth Edition (DSM-5) criteria were used to diagnose children with ASD. The same scale was also used to clarify the severity level of ASD, and a paediatric psychiatrist performed the clinical evaluation. In addition, the Autism Behaviour Checklist (ABC) as a tool for ASD assessment was also applied. ABC is a list of 57 questions assessing the behaviours and symptoms of ASD and rating their scale for children above 3 years old. The ABC consists of questions in five categories: Sensory, Relating, Body and Object Use, Language and Social, and Self-Help skills.

Typically developing sex-, BMI-, and age-matched children with no known health problems were enrolled to form the control group. All control participants were determined healthy based on a questionnaire, standard clinical evaluation, and urinary dipstick test.

Having another neuropsychiatric disorder, chronic diseases such as diabetes, hypertension, etc., abnormal urine test results, complaints related to the urinary system, and using any medication were determined as exclusion criteria. In addition, the presence of leucocytes, protein, blood, glucose, bilirubin, nitrite, and urine pH being less than 5 or more than 7, were considered abnormal after the urine test result.

### Spectral data collection

All the spectra were collected using a Thermo Scientific Nicolet 6700 FTIR spectrometer with a diamond attenuated total reflection (ATR, ConcentratIR2, Harrick) accessory with ten internal reflections. A deuterated triglycine sulphate (DTGS) detector was used for measurements. First, the diamond surface was cleaned with ethanol and rinsed with water. It was then air-dried for 2 min before recording the background interferogram. A frozen urine sample was thawed at room temperature and stirred using a vortex. Five microliters of the urine sample were pipetted onto the diamond surface and dried for 15 min by a gentle stream of N_2_ gas prior to data collection to remove the excess water. All samples were recorded in the 4000–600/cm wavenumber range, and each spectrum was obtained by averaging 128 interferograms for a final resolution of 4/cm. A total of 128 scans with the DTGS detector take about 2 min to complete. Each sample was deposited and measured three times to eliminate pipetting errors and experimental variations. The average of the triplet measurement from each sample was used for further analysis. The Thermo Scientific OMNIC Spectrometer software, version 8.2.388, was used for spectra collection. The spectrometer was constantly purged with N_2_ during measurements to eliminate atmospheric variations in the water vapor.

For the peak analysis results shown in Figs. 4 and 6, integration limits and baseline points are listed in Table 1. Spectra were not pre-processed before integration; however, limits of integration were chosen to exclude the contribution of other urine components, and a suitable baseline was chosen to eliminate the baseline differences among spectra. In order to see the differences in the analysis results due to different pre-processing methods, the spectra were also area normalized before integrating the 3000–2600/cm region. Normalization to the urea band at 1605/cm was also tested in that regard.

**Table 1:** List of integrated peaks, integration limits, and baseline points.

Peak (1/cm)	Integration limits (1/cm)	Baseline points (1/cm)
780	800–765	Straight line between 800 and 765
930	963–890	Straight line between 963 and 890
992	1010–970	Straight line between 1010 and 970
3000–2600	3000–2600	Straight horizontal line at 3999
3200	3250–3150	Straight horizontal line at 3275
3335	3400–3280	Straight horizontal line at 3275

### Statistical analysis

All statistical analyses and visualizations were performed using OriginPro 2017 SR2 and SPPS statistics for Windows, version 22.0 (IBM Corp., NY, USA). Shapiro–Wilk test and Kolmogrov–Smirnov with histogram curves were used to investigate the normality of the distribution. The numerical data were presented as mean value ± standard deviation (SD) if normally distributed; if not, they were presented as median value and inter-quartile range (IQR). An independent *t*-test was used to analyze the differences among normally distributed numerical parameters, while a Mann–Whitney *U* test was used for abnormally distributed ones. A Chi-Square test was performed for the comparison of categorical data. One-way ANOVA and Kruskal Wallis tests were used for the analysis of the parametric and nonparametric data, respectively, for comparing three or more subgroups. Data obtained by integrating spectra were also analyzed by the One-way ANOVA followed by Fisher’s Least Significant Difference (LSD) post hoc test to compare the means. This test determined whether the difference between two means (ASD+ and TD) was statistically difference. In addition, Levene’s test was applied to evaluate the homogeneity of variance. The results were considered statistically significant if the p value was detected as < 0.05.

In this study, the principal component analysis (PCA) was performed. This unsupervised technique is a projection method that progressively searches for directions in multivariate space that provide the best fit to the data distribution^38,39^. It is a dimensionality reduction technique commonly used in data analysis and machine learning when dealing with high dimensional data, where the number of features is much larger than the number of samples^38^. PCA was used to determine any relationships among the multiple data, visualize variable influence and data distribution, compare data groups, cluster, and interpret data. To perform a change of basis on the data, PCA uses the principal components (PC). Score plots were used for data visualization, and the loading plots explained which variables contribute the most to the positioning of the samples on the score plot.

### Artificial urine preparation

An artificial urine (AU) solution was prepared according to the protocol and ingredients published previously^31^. To test the spectral contribution of urea, twice the normal amount was used in preparing the solution. Next, the spectrum of this new AU with exaggerated urea was compared with that of the AU at the normal amount. The difference between these two spectra was used to locate the spectral regions and peaks that urea affects. The same procedure was repeated with uric acid, phosphate, ammonium, and sulphate. MgSO_4_ and NH_4_Cl were used as the source of sulphate and ammonium, respectively. A combination of Na_2_HPO_4_ and NaH_2_PO_4_ was used as the phosphate source for preserving the pH. The difference spectra are presented in Fig. 5.

### Ethics approval and consent to participate

This study was approved by the Atilim University Human Studies Ethics Committee (Ref. Number: 59394181-604.01.01-8509). The participants’ parents signed an informed consent form stating that they volunteered and that the data obtained would be published anonymously.

## Results and discussion

In this study, the infrared spectra of urine from 26 children diagnosed with autism spectrum disorder (ASD+) are compared to those of 26 healthy, typically developing children. Both ASD+ (n = 26) and control (n = 26) groups were the same in terms of gender. The age (42.2 ± 9.1 months in ASD + , 44.7 ± 8.3 months in controls; p = 0.585) and BMI (16.3 ± 2.9 kg/m^2^ in ASD + and 15.7 ± 1.9 kg/m^2^, in controls; p = 0.285) were also statistically similar in these groups. Although it was not statistically significant, the parameter ‘sign of mental illness in a relative’ is slightly more in the ASD+ group (6/26, 23.1%) compared to that in controls (1/26, 3.8%) (p = 0.086). Family income is higher in controls than in ASD+, and it is different with statistical significance (p < 0.0001). The majority (14/26, 55.8%) of ASD+ patients’ family income is in the range of 2500–5000 Turkish Lira (TL) ($300–$600), while 15 of 26 (57.7%) in controls are in 10,000–20,000 TL ($900–$1800) range. One-way ANOVA test revealed that only the parameter ‘body and object use’ was different statistically (p = 0.037). Post hoc analysis showed that this difference was due to the ASD Level 2 group. Other ABC score

**Table 2:** Demographic and clinical data of the ASD + and the control group participants.

Demographic characteristics					
Parameters			ASD + (n = 26)	Control (n = 26)	p value
Age (months) (mean ± SD)			42.2 ± 9.1	44.7 ± 8.3	0.585
Gender					1.000*
Male; n (%)			23(88.5)	23(88.5)	
Female; n(%)			3 (11.5)	3(11.5)	
BMI (mean ± SD)			16.3 ± 2.9	15.7 ± 1.9	0.285
Sign of mental illness in a relative					0.086*
Yes, n(%)			6 (23.1)	1(3.8)	
No, n(%)			20 (76.9)	25(96.2)	
Special education (no/yes)			15/11	−	−
Family income (1–5) (median, IQR)			1.5, 1–2	3, 2–4	< 0.0001**
0–2500 TL (0–$300) (1)			3 (11.5)	2 (7.7)	
2500–5000 TL ($300–$600) (2)			14 (53.8)	3 (11.5)	
5000–10,000 TL ($600–$900) (3)			5 (19.2)	3 (11.5)	
10,000–20,000 TL ($900–$1800) (4)			4 (15.4)	15 (57.7)	
> 20,000 TL (> $1800) (5)			0	3 (11.5)	

Clinical assessment score for ASD + participants					
Autism behaviour checklist (ABC)	ASD level 1 mean (± SD)	ASD level 2 mean (± SD)	ASD level 3 mean (± SD)	ASD total mean (± SD)	p value
Sensory	6.7 (4.3)	7.4(4.3)	9.8(5.3)	7.7(4.3)	0.576***
Relating	10.0 (8.1)	12.9(8.2)	18.2(8.0)	13.3(8.2)	0.358 ***
Body and object use	14.5 (7.0)	9.7(6.1)	19.5(7.8)	12.1(7.4)	0.037***
Language	10.5 (5.4)	11.3(5.2)	6.4(5.3)	10.2(5.4)	0.215***
Social and self-help	10.2 (5.4)	8.8(5.6)	6.6(6.1)	8.6(5.4)	0.601***
ABC total score	52.0 (15.4)	50.1 (16.6)	63.8 (18.4)	52.7 (18.4)	0.428***

Significant values in bold.

Student T test, *Chi Square test, **Mann–Whithey *U* test, ***One way ANOVA.

*TL* Turkish Lira, *$* US Dollar. The hunger threshold was $340, and the poverty threshold was $1100 during the time data was collected^40^.

s were similar statistically. The data are presented briefly in Table 2.

We found that the family income was higher and significantly different in TD controls. The studies relating ASD to family income indicate that its prevalence is higher in families with high household incomes^41^. It is emphasized that the difference may also be due to the delay or lack of diagnosis in low-income households. In this study, firstly, the ASD+ group was formed, and then the TD children who were admitted paediatric psychiatry clinic of the tertiary university hospital were assigned to the control group. Thus, families who take their children to regular paediatric evaluations can be expected to be in a better socio-economic status since they are mostly the families with high awareness and education levels. Irrespective of the reason, family income levels may affect the diet habits, its content, educational status, and the environmental factors of children^42,43^. In addition, some of the possible mechanisms discussed in the literature about ASD are related to diet and microbiota^44^. To see the effect of family income difference on the urine spectra, the spectra of TD children from income groups 1 and 5 are compared (Supporting Information Fig. S1). The spectral difference between these two groups is minute compared to that observed between ASD+ and TD group children. Possible reasons for this observation are discussed in Supporting Information. Therefore, presented spectral data indicate disease-related differences.

Other clinical data of the participants were obtained using the urinary dipstick test. The average value of specific gravity (SG) is 1.02 (min: 1.005, max: 1.030, SD: 0.009) for ASD+ children and 1.028 (min: 1.020, max: 1.030, SD: 0.009) for the control group (p = 0.163). The average urinary pH is 6.3 (min: 5.5, max: 7.0, SD: 0.85) for ASD+ children and 5.6 (min: 5.0, max: 6.0, SD: 0.38) for the control group (p = 0.009).

### FTIR spectra of urine

**Figure 1:**
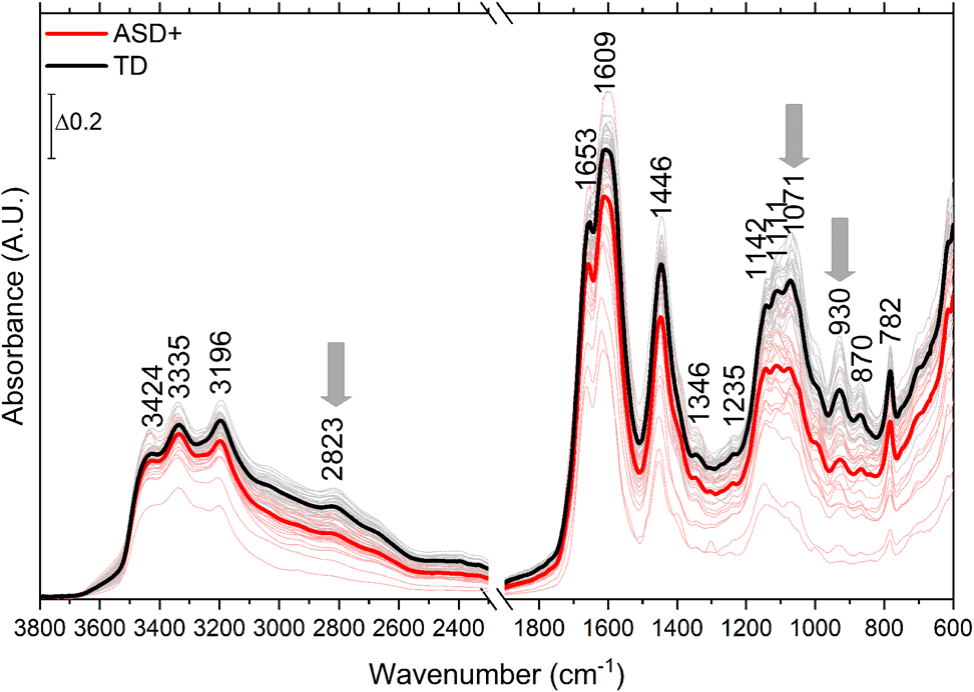
Recorded infrared spectra of all ASD+ (pale red) and TD (gray) children. The average spectrum of ASD+ is shown in red, and that of TD is in black (thick lines). The spectra shown are not pre-processed.

Figure 1 shows all the collected spectra overlaid with the average spectrum of all ASD + children and the average of controls. As can be seen, the two average spectra are comparable in terms of the general spectral profile and prominent peak positions. Nevertheless, there are marked differences that are common among all pair-wise comparisons. The two average spectra are significantly different at the 0.05 level based on the Mann–Whitney *U* test (Z = ‒8.6, U = 5.48 × 10^6^).

**Figure 2:**
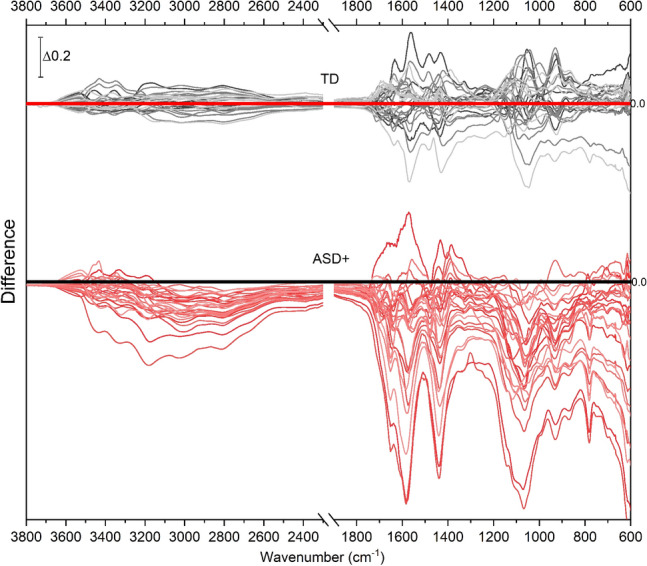
The difference spectra obtained by subtracting the spectrum of each participant from the calculated average spectrum of the control group. The subtraction is formulated as [S_participant_ ‒ S_average control_], where S stands for the spectrum. The results of the control group are compiled in the upper panel (gray) and those of the ASD+ group in the lower panel (red).

When individual spectra from the ASD+ group are pair-wise compared to their controls, it is observed that almost all the spectra from ASD+ children have lower absorbance at all points than those from their control children (data not shown). However, the deviation of each spectrum with respect to its control is not uniform, being high at some points as 1071 and 930/cm compared to others. To demonstrate the absorbance differences between the spectra of ASD+ and control children, the difference spectrum of each ASD+ participant from the average spectrum of controls is shown in Fig. 2. The positive peaks indicate higher absorbance with respect to the average control, whereas the negative peaks indicate lesser absorbance. In a previous study, it has been shown that the spectra of the healthy children population show the least intensity variations compared to other age groups^32^. In the same study, the urine spectra collected from 41 children aged 3–10 were shown to be similar despite the wide age range and the difference in gender, dietary habits, and their parents’ socio-economic status. In this study, the spectra of the control children are also very similar in intensity (Fig. 1-gray lines). The intensities of the difference spectra are particularly low in the high wavenumber region for these children (Fig. 2-upper panel). On the other hand, the absorbance difference from the average spectrum of controls is substantial for ASD+ children on the same scale (Fig. 2-lower panel). It can be observed that most ASD+ urine spectra have lower absorbance than the average spectrum of controls. It can be due to the lack of some urine components excreted consistently by ASD+ children. Although the low-frequency region (1800–600/cm) shows this difference more dramatically, in essence, the high-frequency region (4000–2400/cm) is more interesting since the control group has the least variation in the same region. Thus, the high-wavenumber region is further analysed in an attempt to quantify and explain the observed deviation.

**Figure 3:**
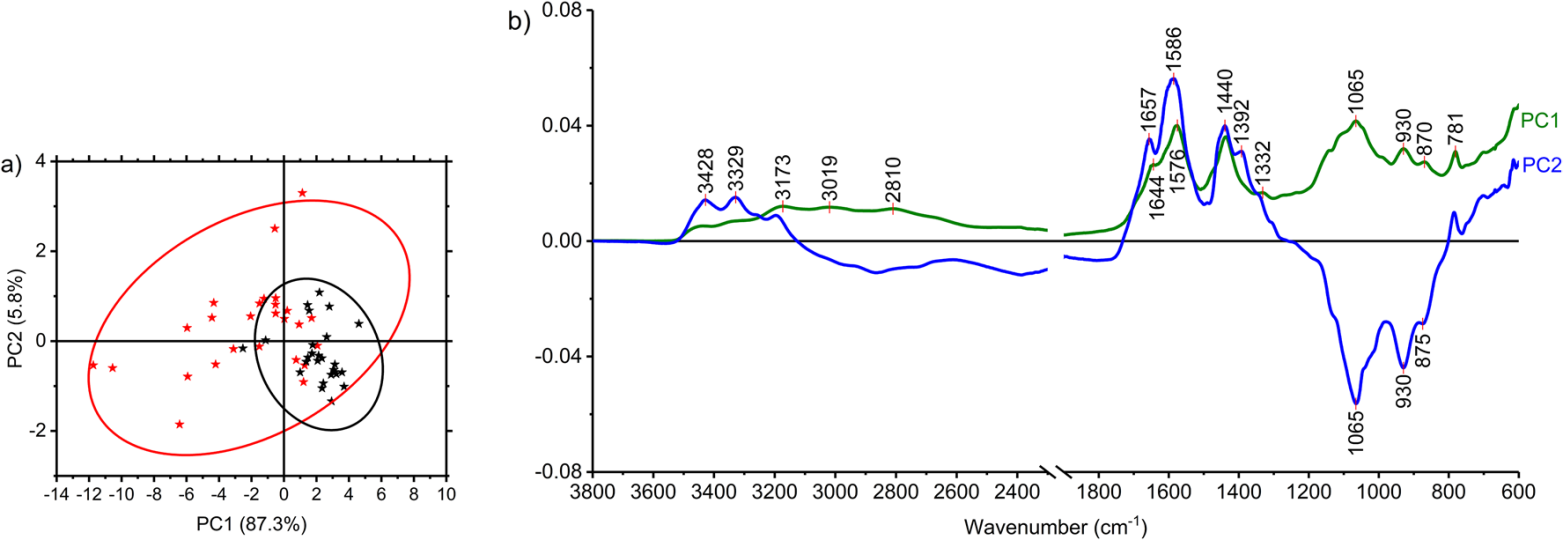
PCA scores (**a**) and PCA loading plots (**b**) of ASD (+) and TD children group for the 3800–600/cm region.

The PCA is performed to determine any relationships among the two groups using the entire spectral region. Figure 3a and b present a two-dimensional PCA scatter plot and the loadings plot of PC1 and PC2 as a function of wavenumber for the 3800–600/cm region, respectively. The score plots present the distribution of ASD+ (red stars) and controls (black stars). Most of the controls are distinguished from ASD+ along the PC1 axis. The PC1 accounts for 87.3% while PC2 accounts for 5.8% of the total variance, both within 95% confidence ellipses. The scores of controls lie on the positive side of the PC1 scores, but most ASD+ are on the negative side. The ASD+ samples show a wider distribution than the controls.

The positive and negative peaks can be observed in the loading plots. The positive peaks indicate the positive score values due to increased absorbance in the spectra. In contrast, we can relate the negative loading values to the increased absorbance in the spectra of the negative score values^45^. A loading plot is used to visualize the relationship between variables and PCs extracted from a dataset. Although PCA does not show a net separation of ASD+ data from that of TD children, it identifies the bands and regions that contributed to the partial dissociation observed in the score plot. The absorbance differences at 781, 930, 1065, 2810, and 3019/cm contributed to clustering the two groups. While the peak at 781/cm can be mainly attributed to uric acid, others are affected by multiple urine components, namely phosphate, sulphate, N–H groups, and urea^31^. Each of these components is discussed separately in the following sections.

### A closer look at the 3000–2600/cm region

**Figure 4:**
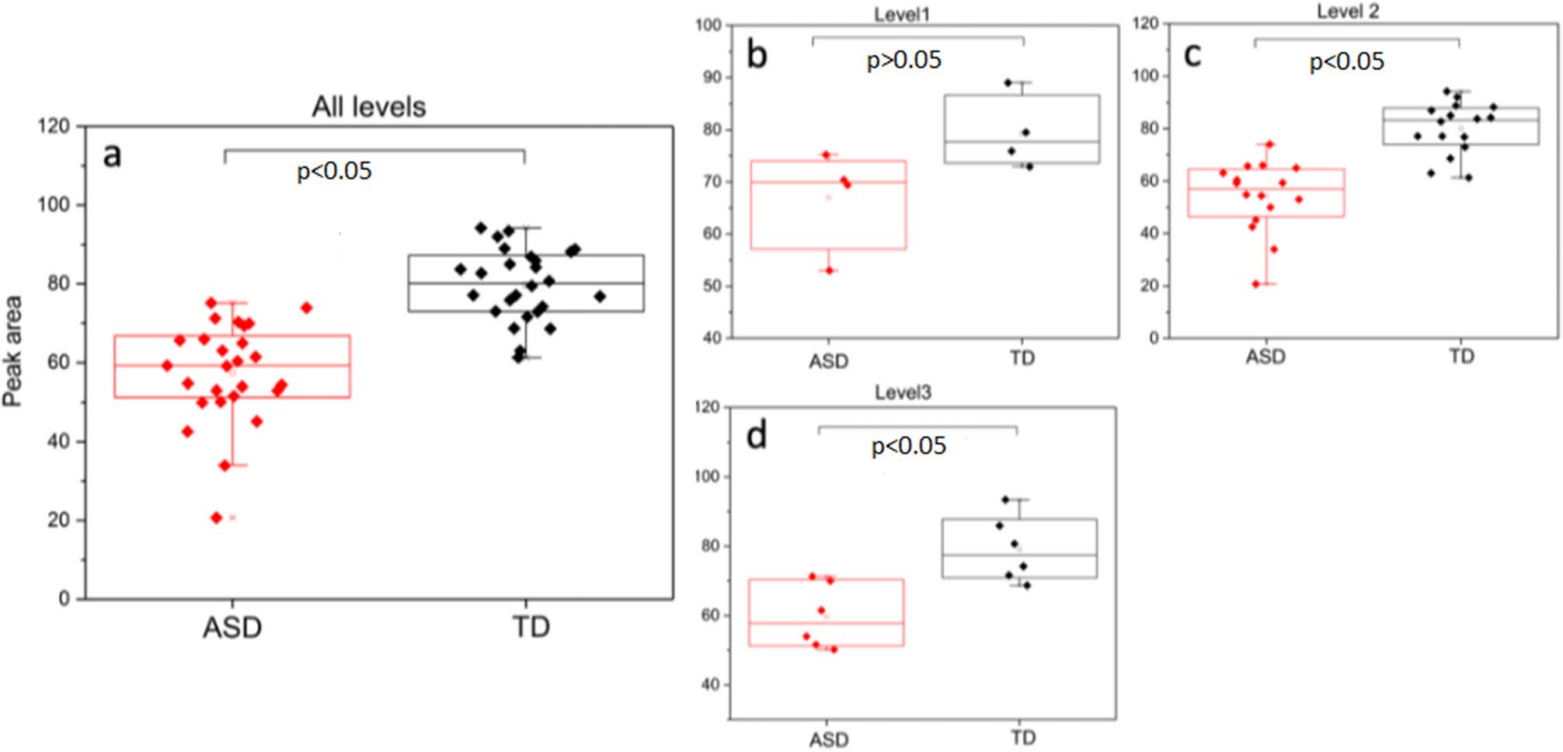
The mean values of the peak area of the 3000–2600/cm region for ASD+ and TD children (**a**). A similar comparison is also shown for each ASD level with respect to their controls in (**b**), (**c**), and (**d**) for ASD level 1, level 2, and level 3, respectively. See “Materials and Methods” for statistical details.

In the high-frequency region, ASD+ children have lower absorbance with respect to their controls in the 3000–2600/cm region (Figs. 1 and 2). The absorbance difference is more dramatic in children with ASD severity levels 2 and 3. In order to quantify the absorbance difference, this region is integrated using raw spectra to find the area under the curve (Table 1). The results are tabulated in Table S1 (Supporting Information). The spectra of the ASD+ level 3 children show 24% less absorbance with respect to controls, while level 2 children show 29% less absorbance in the same region. Although the ASD+ level 1 children also show the same trend (16% less), the difference is less in comparison to levels 2 and 3. Figure 4 compares the mean of the peak area for all ASD levels using ANOVA with Fisher’s LSD and Levene’s test. The results show that the population variances are similar for all ASD levels, but the population means are significantly different (Fig. 4a). The difference between ASD+ and TD groups is significant for levels 2 and 3, except level 1 (Fig. 4b–d).

Interestingly, the average area of the 3000–2600/cm region in the TD children, compared to that of the ASD+ group at each level, is nearly the same (Table S1). This integrated region shows slight variance among healthy children, as previously shown in a study with 41 children aged 3–10^32^. In the same study, the two genders showed no difference within the same age group in the high-frequency region.

Two other common normalization methods in the literature, namely, the area normalization and the min–max normalization (1605/cm-urea normalization), are also tested for the same area calculation. The results of these tests are summarized in Table 3. The area of the 3000–2600/cm region is consistently less in all ASD+ children with respect to their controls. Different normalization methods reduced, but did not eliminate in full, the absorbance difference between the two groups. Therefore, it can be suggested that possible urine concentration differences among the children are not a factor in the observed results.

**Table 3:** The percentage difference of the area of the 3000–2600/cm region between ASD+ and TD children using raw spectra (no pre-processing applied), area normalized spectra, and min–max normalized spectra (normalized to 1605/cm-urea band). Area values are higher in the control group spectra than in the spectra of ASD + children.

ASD severity level	Raw spectra	Min–max normalization	Area normalization
1	15.5%	9.7%	4.1%
2	29.4%	24.1%	12%
3	24.3%	19.1%	9.6%

### Origin of the absorbance difference in the 3000–2600/cm region

**Figure 5:**
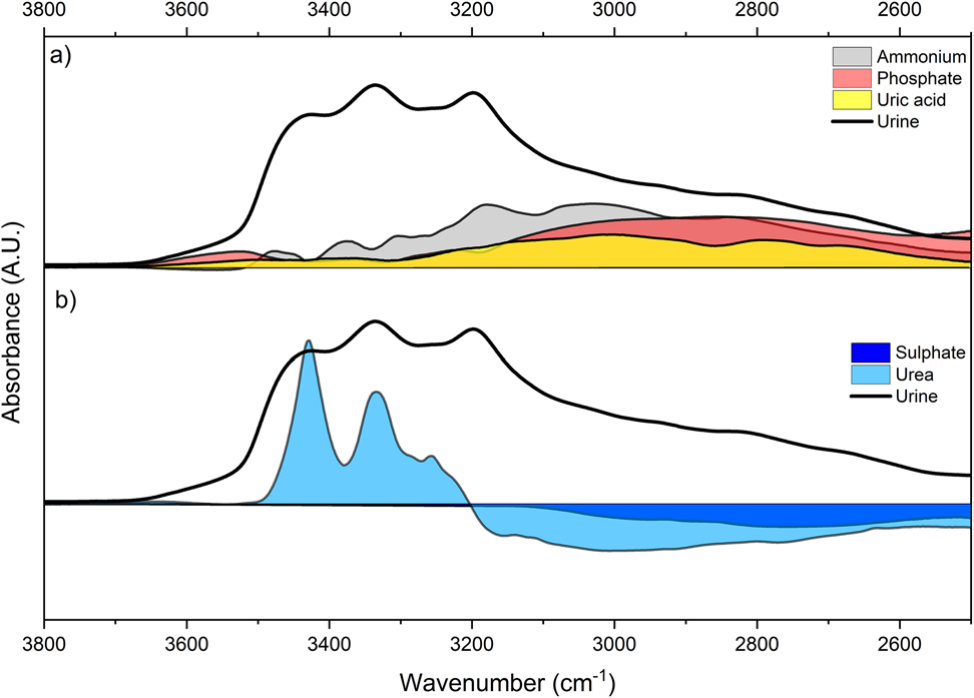
Spectral contribution of increased ammonium, phosphate, uric acid, sulphate, and urea in the high wavenumber region. (The spectra are obtained using artificial urine solutions, as explained in the Materials and Methods section).

The source(s) of this difference in absorbance between the ASD+ and TD children is not certain; however, based on the individual urine component measurements in previous studies^31^, it is possible to comment on the probable sources. Less-than-normal levels of uric acid, phosphate groups, ammonium (${NH}_{4}^{+}$), or more-than-normal levels of urea and sulphate groups can be listed as probable causes. To test the contribution of these components, an artificial urine solution is prepared^31^. The spectrum of this artificial urine is compared to that of another solution, including twice the uric acid than normal, so that the spectral contribution of uric acid and its marker bands can be observed. The difference between these two spectra for the high-frequency region is given in Fig. 5. It can be seen that the increased amount of uric acid has a positive contribution to the 3300–2500/cm region of the spectrum. The same procedure is repeated for ammonium and phosphate separately, each of which component positively contributes to the absorbance in the same region. On the other hand, increased urea or sulphate decreases the absorbance of the same region and, thus, has a negative effect.

### Examination of spectra in terms of ammonium, phosphate, uric acid, sulphate, and urea

These components, while affecting the absorbance in the high-frequency region, also have other peaks in the low-frequency region. In the subsequent sections, each of these components is discussed separately in terms of the levels detected in our cohort, their importance, and their possible relation with ASD using previous studies. For each urine component discussed below, the effect of dietary habits is not discussed since FTIR spectroscopy is less sensitive to slight changes in the urinary level of these components compared to biochemical analyses. Additionally, dietary choices are relatively healthier and less variant among children of age 3 to 5 compared to adults. In fact, the distribution of variation seen within the TD group can be attributed to different dietary habits. The degree of differences among the spectra of TD children is far less than the observed changes between the two groups in this study, which mostly rules out the effect of diet.

### Phosphate group

**Figure 6:**
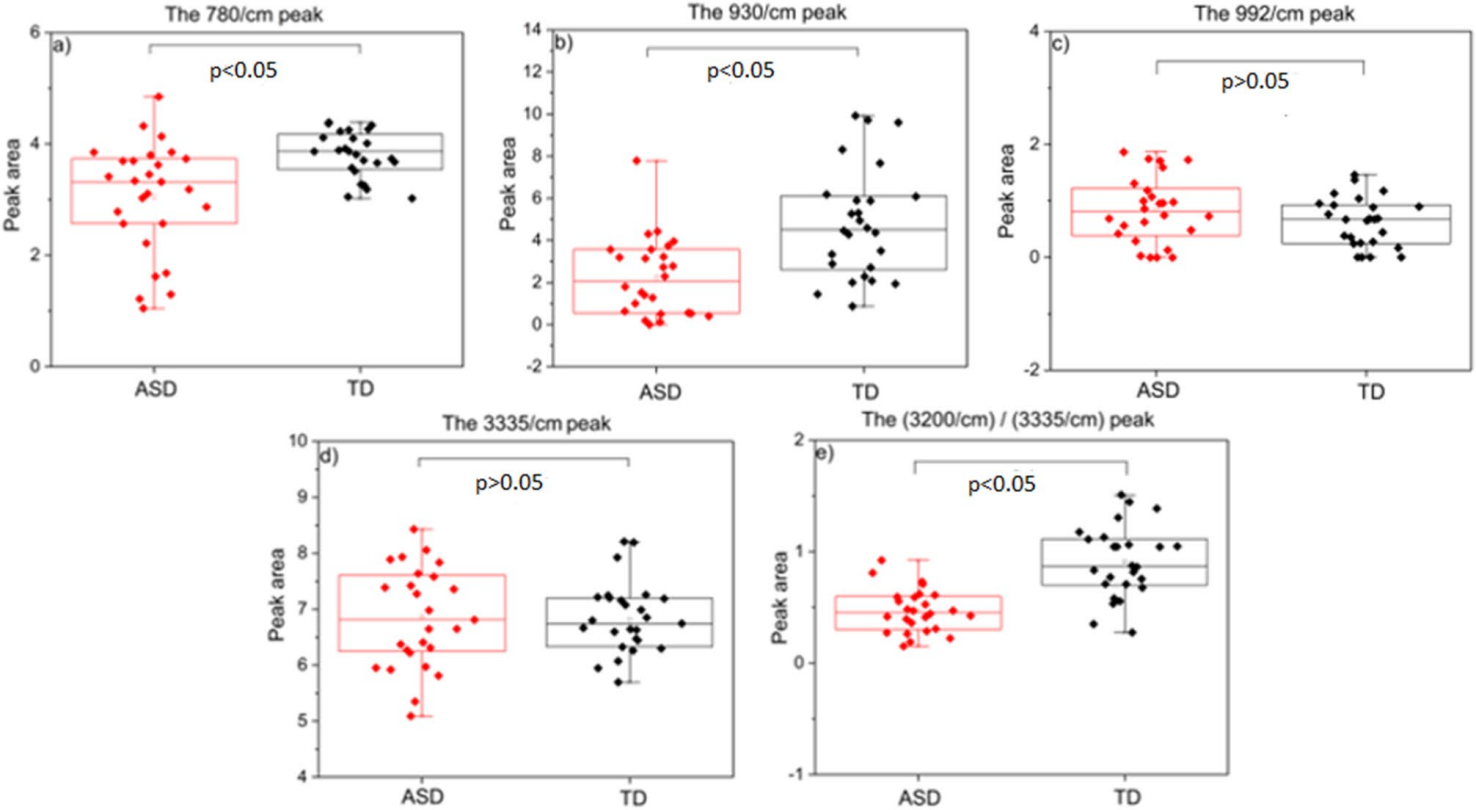
Comparison of selected peak areas between the TD group and the ASD+ group of children. Each peak represents a separate urine component, as discussed in the relevant sections. Statistical analysis was done by one-way ANOVA followed by Fisher’s post hoc test. Integration limits and baseline choices are given in Table 1.

The urinary phosphate levels can be followed by peaks at ~ 1050 and 930/cm. Upon second derivative calculations, the peak at 1050/cm is seen to overlap highly with those of the sulphate groups and the urea (data not shown). Thus, the peak at 930/cm, attributed to the P-OH bending mode, is used to track changes in the amount of phosphate excreted in the urine^31^. The calculated area of this peak is compared between the two groups in Fig. 6b. Although the diversity of peak areas is similar in both groups, the mean value is observed to be less in ASD+ children compared to that of the control group. The difference in the means is significant at the 0.05 level. Therefore, it can be concluded that low urinary phosphate levels have contributed to the lesser absorbance in the 3000–2600/cm region of the spectra of ASD+ children.

It is well known that phosphate (Pi) has essential roles in the intracellular compartment and the skeleton. Many factors may affect its absorption and reabsorption in the kidneys, such as the amount ingested with the diet, parathyroid hormone, other hormones (insulin, growth hormone/insulin-like growth factor 1, calcitonin, glucocorticoids, atrial natriuretic peptide, phosphatonin, prostaglandins, stanniocalcin, etc.), and non-hormonal mechanisms (fasting, plasma calcium, acid–base status, volume status, and medication)^46^. The urinary phosphate excretion is also linked to vitamin D levels, which depend on calcium and phosphate for bone formation. Some ASD+ children were also reported to have vitamin D deficiency, and the amount of serum 25 (OH) D_3_ was significantly low^47,48^. In another study, urinary hippurate and phosphate levels were shown to be low in ASD+ children compared to their controls (n = 26 ASD+, 24 TD)^20^. Another previous study also showed lower amounts of phosphoric acid, another phosphorus source in urine, in ASD+ children compared to controls (n = 156 ASD+, 64 TD)^13^. The results of this study are in line with the previous findings regarding the phosphate.

### Uric acid

FTIR has several signatures for uric acid; however, many are overlapped by stronger urea absorptions. A small peak at 1345/cm or at 780/cm can be used to monitor uric acid changes. The 780/cm peak is more isolated from the contribution of other urine components, and thus is preferred over the 1345/cm peak, although the analysis of both gives similar results (data not shown). The population variances are significantly different when the 780-peak area is calculated and compared (Fig. 6a). The control group displays close results, but the ASD+ group shows a diversity. The difference between the mean of the peak areas is also significant at the 0.05 level. The 780-peak is less intense in the ASD+ group, meaning lower uric acid levels in the same group than in the controls. As a result, low uric acid levels could have contributed to the absorbance difference measured at the 3000–2600/cm region. In ASD, the level of antioxidants excreted in urine was shown to be significantly lower than TD children^49^. The same study also showed that the amount of urinary uric acid excreted by children with low-functioning autism (1.23 g/day), medium-functioning autism (0.99 g/day), and high-functioning autism (0.69 g/day) was higher than that by TD children (0.62 g/day). However, two other studies showed that the urinary uric acid level in ASD + children is low compared to the TD children^4,22^.

### Sulphate group

Many factors influence the sulphate levels in urine, such as age, gender, pregnancy, medication, disorders, etc. In the normal state, the sulphate excretion rates are lower in children than in adults, and very low in infants, implying the conservation of sulphate during growth^50,51.^ Sulphate has an inverse effect in the high-frequency region of the spectrum; namely, as the urinary sulphate level increases, the area under the curve decreases in the high-frequency region. Urinary sulphate levels can be followed by the intensity changes at 992 and 1090/cm that are tentatively assigned to $\text{R-S}{\text{O}}_{2}^{-}$ and S=O, respectively. The relatively more isolated peak at 992/cm is preferred for comparing two groups in terms of the sulphate levels. Figure 6c shows the 992/cm peak area changes using One-way ANOVA for ASD+ and TD. The results show that the population variances and the difference between the means are not significantly different. Therefore, urinary sulphate is not considered as one of the contributors to the observed difference in the high-frequency region. Contrary to the findings in this cohort, high or low sulphate levels have been associated with ASD in previous studies.

Renal sulphate wasting due to mutations in the sulphate reabsorbing carrier protein is found in some cases of autism^52,53^. The low urinary levels of 4-cresol sulphate, hippurate, and creatine in ASD+ children were documented previously^28^. On the other hand, in other studies, the urinary indoxyl sulphate levels were significantly higher in ASD+ children compared to controls^6,17,27^. Another research showed that ASD+ children (average age 7.6) had high urinary sulphate, thiosulphate, and sulphite^54^. A study conducted in the U.S. documented lower sulphate levels in water samples taken from regions where autism is diagnosed more frequently^55^.

### Ammonium

The urinary ammonium ion (NH_4_^+^) indicates eliminated free hydrogen ions (protons) to balance kidney acidosis. Most of its overall concentration comes from tubular cells, especially the proximal tubular cells—and not due to glomerular filtration rate—as opposed to sulphate. Various factors may affect the activity of the NH_3_-producing enzyme glutaminase, and these factors can be summarized under two categories: systemic and intra-renal. The systemic factors are low levels of plasma bicarbonate, low plasma pH, and the action of adrenal steroids. The intra-renal factors are intracellular potassium deficiency and low intracellular pH. Under the influence of one or more of these factors, glutamine is cleaved by glutaminase to glutamate and NH_3_ in tubular cells. NH_3_ reacts with the H^+^ ions to form NH_4_ in the intra-tubular fluid, thereby neutralizing the H^+^ ions^56^.

The N–H vibrational modes absorb strongly in the high-frequency region, whereas only small changes can be observed in the low-frequency region, particularly at 1660, 1430, and 930/cm. Since the first two of these peaks overlap with strong urea absorptions and the latter is dominated by the phosphate group absorptions, another peak at 3200/cm in the high-frequency region had to be selected. However, urea also has a contribution to the same position. Thus, changes in urea absorption, measured at 3335/cm, are used to eliminate its contribution to the 3200/cm peak. The ratio of the calculated peak areas, i.e., 3200/3335/cm, shows that the ASD+ children have lower values compared to the controls (Fig. 6e). The difference between the means and the population variances are significantly different at the 0.05 level. Low ammonium levels due to the neutral-to-alkaline pH of urine can contribute to the observed smaller area in the 3000–2600/cm region. The average urine pH of our ASD+ children (6.3) is slightly more than that of the TD children (5.6). Although this slight difference in urinary pH could be the cause of the presented spectral findings, to the best of our knowledge, there is no other study showing the same urinary pH trend to back up the current hypothesis. The urea/ornithine cycle is a sequential multiple biochemical reaction that ends with the production of urea (NH_2_)_2_CO from ammonia (NH_3_) in the mitochondria of liver cells. The literature shows that there is a relation between urea cycle disturbances and mental retardation^57^. According to a previous study, accumulated ammonia produced by *H. pylori* causes the observation of elevated serum ammonia among ASD+ children^58^. Several studies have shown that the serum ammonium level is high in ASD+ children with respect to controls^59–61^.

### Urea

An increase in urea can be directly followed in the spectrum by the growing intensity in the 3420, 3335, and 1609/cm peaks. However, urea has the opposite effect in the 3000–2600/cm region; as the amount increases, the region intensity decreases. Urea (nitrogen compounds) is the end-product of amino acid metabolism; its level in the urine is directly proportional to protein intake. A protein-rich diet tends to decrease the absorbance in the 3000–2600/cm region; however, no such dietary habit was reported in the questionnaires by the families of ASD+ children. In fact, when 3335-peak areas are compared, the difference between the means is not significant and the population variances are similar (Fig. 6d). Therefore, it is suggested that urea does not contribute to the lower intensities observed in the 3000–2600/cm region in the ASD+ group.

### Other possible urine components

Previous studies with autistic children documented low levels of phenylactic acid, aconitic acid, 3-oxoglutaric acid, vitamin B6, and kynurenine^6,13^. These components also have large-base and low-amplitude absorbances in the 2600–3000/cm region of the spectrum^62^. Each component listed in the previous sections has typical concentrations ranging from µmol to mmol in the average urine^26^. Therefore, the spectroscopic results provided in Figs. 1 and 2 cannot be the results of one component alone; rather, they are the consequence of a probable common habit or metabolism among autistic children, eventually altering several components within the urine.

## Conclusion

In this study, 26 children aged 3–5 years, newly diagnosed with ASD+, using no medications are studied, and the usability of ATR-FTIR is investigated. The high-frequency region of the urine FTIR spectrum is rarely used in literature due to its complex nature of overlapping bands. This study shows that it can be used as a single window that shows the net effect of multiple urine constituents at once that are important for ASD physiology. It is shown that the infrared spectra of ASD+ children display lower absorbance in the 3000–2600/cm region of the spectrum compared to TD children. PCA is used to identify the peaks that contributed to the dissociation of two groups. It clusters the healthy children group with 87.3% variance along the PC1 axis. The average spectral area of the high-frequency region is 25% less in ASD+ severity level 3 children, 29% less in ASD+ level 2 children, and 16% less in ASD+ level 1 children compared to that of TD children. Level 1 ASD+ children are more challenging to detect with this method since some of the diagnosed children have the same urine spectrum as their controls. Urinary components as possible sources of this outcome are discussed within the manuscript. In previous studies, most of these components have been detected and linked with autism. Spectroscopic results suggest that lower-than-normal levels of uric acid, ammonium, and phosphate groups may have contributed to the observed results in this study. No evidence is found for the contribution of urea and sulphate groups in this study. Also, the average urinary pH is slightly shifted towards neutral in ASD+ children. The nature of the relation (result vs. cause) between these components and the physiology of autism remains an unanswered question.

Studying urine composition component-by-component is not practical, particularly from a clinical point of view. Instead, infrared spectroscopy offers a general picture of urine composition. It is suggested that a visual comparison of the spectrum with that of a healthy child is enough to know that there may be a problem without a calculation. The physician then at least entertains the possibility of autism during standard follow-ups and can take steps, ask questions, or suggest another evaluation. For this disorder, the etiology of which we still do not know and for which there is no biomarker yet, every trait that will contribute to the referral process to child psychiatry is valuable. Even if FTIR does not make a differential diagnosis, it is enough to raise suspicions.

Based on the experience gained in this study, a reference control spectrum to be used in such comparisons should be constructed (by averaging) using the spectra of children of the same age and gender. Thus, it is proposed that the average spectrum constructed from a pool of 3-year-old boys can be used as the negative of another 3-year-old boy. For the evaluation of a 5-year-old girl, a pool of 5-year-old girls can be used.

In order to prove the usefulness of this spectroscopic method for preventive medicine, larger pools of ASD+ and healthy children should be studied. The lack of blood biochemistry results prevented further clinical interpretation of the urine results obtained in this study. For instance, blood gas analysis could have been performed for a more accurate evaluation of urine pH results. The role of ethnicity should also be studied to discuss the possibility of a universal spectrum pool. It is also unknown whether the observed spectral difference is ASD-specific. Other neurodevelopmental disorders like intellectual disability, global developmental delay, attention-deficit/hyperactivity disorder, motor and tic disorders, specific learning disorders, and communication disorders should be studied in this regard.

### Supplementary Information

Supplementary Information.

## Data Availability

The datasets used and/or analyzed during the current study are available from the corresponding author upon reasonable request.
