# Author Correction: Using urine FTIR spectra to screen autism spectrum disorder

**DOI:** 10.1038/s41598-024-57148-1

**Published:** 2024-03-21

**Authors:** Neslihan Sarigul, Leyla Bozatli, Ilhan Kurultak, Filiz Korkmaz

**Affiliations:** 1 Institute of Nuclear Science, Hacettepe University, Ankara, Turkey; 2 Department of Child and Adolescent Psychiatry, Faculty of Medicine, Trakya University, Edirne, Turkey; 3 Department of Nephrology, Faculty of Medicine, Trakya University, Edirne, Turkey; 4 Biophysics Laboratory, Faculty of Engineering, Atilim University, Ankara, Turkey

Correction to: *Scientific Reports* 10.1038/s41598-023-46507-z, published online 09 November 2023

The original version of this Article contained an error in Table 2, where “Male” and “Female” rows in Parameters were inadvertently switched.

The original Table 2 and accompanying legend appear below.

**Table 2:** Demographic and clinical data of the ASD + and the control group participants.

Demographic characteristics					
Parameters			ASD + (n = 26)	Control (n = 26)	p value
Age (months) (mean ± SD)			42.2 ± 9.1	44.7 ± 8.3	0.585
Gender					1.000*
Female; n (%)			23(88.5)	23(88.5)	
Male; n(%)			3 (11.5)	3(11.5)	
BMI (mean ± SD)			16.3 ± 2.9	15.7 ± 1.9	0.285
Sign of mental illness in a relative					0.086*
Yes, n(%)			6 (23.1)	1(3.8)	
No, n(%)			20 (76.9)	25(96.2)	
Special education (no/yes)			15/11	–	–
Family income (1–5) (median, IQR)			1.5, 1–2	3, 2–4	< 0.0001**
0–2500 TL (0-$300) (1)			3 (11.5)	2 (7.7)	
2500–5000 TL ($300–$600) (2)			14 (53.8)	3 (11.5)	
5000–10,000 TL ($600—$900) (3)			5 (19.2)	3 (11.5)	
10,000–20,000 TL ($900—$1800) (4)			4 (15.4)	15 (57.7)	
> 20,000 TL (> $1800) (5)			0	3 (11.5)	

Clinical assessment score for ASD + participants					
Autism behaviour checklist (ABC)	ASD level 1 mean (± SD)	ASD level 2 mean (± SD)	ASD level 3 mean (± SD)	ASD total mean (± SD)	p value
Sensory	6.7 (4.3)	7.4(4.3)	9.8(5.3)	7.7(4.3)	0.576***
Relating	10.0 (8.1)	12.9(8.2)	18.2(8.0)	13.3(8.2)	0.358 ***
Body and object use	14.5 (7.0)	9.7(6.1)	19.5(7.8)	12.1(7.4)	0.037***
Language	10.5 (5.4)	11.3(5.2)	6.4(5.3)	10.2(5.4)	0.215***
Social and Self-help	10.2 (5.4)	8.8(5.6)	6.6(6.1)	8.6(5.4)	0.601***
ABC Total score	52.0 (15.4)	50.1 (16.6)	63.8 (18.4)	52.7 (18.4)	0.428***

Significant values in bold. Student T test, *Chi Square test, **Mann–Whithey *U* test, ***One way ANOVA. *TL* Turkish Lira, *$* US Dollar. The hunger threshold was $340, and the poverty threshold was $1100 during the time data was collected^40^.

The original Article has been corrected.

