# Supplementary material for: Using urine FTIR spectra to screen autism spectrum disorder
Source: Sci Rep. 2023 Nov 9;13:19466. doi: 10.1038/s41598-023-46507-z (PMC10636094; doi:10.1038/s41598-023-46507-z)
Supplement: Supplementary file 1 — Supplementary Information. [file 41598_2023_46507_MOESM1_ESM.docx]

**Using Urine FTIR Spectra to Screen Autism Spectrum Disorder**

Neslihan Sarigul^1^, Leyla Bozatli^2^, Ilhan Kurultak^3^, Filiz Korkmaz^4^

^1^ Institute of Nuclear Science, Hacettepe University, Ankara, Turkey

^2^ Faculty of Medicine, Department of Child and Adolescent Psychiatry, Trakya University, Edirne, Turkey

^3^ Department of Nephrology, Faculty of Medicine, Trakya University, Edirne, Turkey

^4^ Biophysics Laboratory, Faculty of Engineering, Atilim University, Ankara, Turkey

**SUPPORTING INFORMATION**

The area under the spectrum (integral) for the region between 3000 and 2600 cm^-1^ was calculated using a horizontal line at 3999/cm as the baseline. Smoothing, baseline correction, and other pre-processing methods were not applied to the spectra. Area calculation was performed for all three measurements of the same sample so that an experimental error can be determined by comparing the three results as Error (E)= (Value_Max_-Value_Min_)/2. The average of the three calculation results was recorded as the result of integration in Table S1.

Table S1. The area under the spectrum for the region 3000-2600 cm^-1^ for all children. Raw spectra are used for the given results. The severity levels of ASD+ children are indicated next to the area values.

|  | Area of the 2600-3000 cm^-1^ region | | | | |
| --- | --- | --- | --- | --- | --- |
| Age | **Gender** | **ASD+ children (ASD severity level)** | **TD children** | **p value** |  |
| 3 | Boy | 70.4 ± 1.6 (1) | 79.5 ± 3.9 | <0.0001 |  |
| 3 | Boy | 69.4 ± 2.9 (1) | 72.9 ± 1.3 | <0.0001 |  |
| 4 | Boy | 75.2 ± 4.6 (1) | 89.0 ± 5.9 | <0.0001 |  |
| 4 | Boy | 53.0 ± 0.5 (1) | 75.9 ± 3.9 | <0.0001 |  |
| Average | | 67.0 ± 2.4 | 79.3 ± 3.8 |  |  |
| 3 | Boy | 50.0 ± 5.1 (2) | 73.0 ± 1.2 | <0.0001 |  |
| 4 | Boy | 34.0 ± 5.1 (2) | 61.3 ± 2.6 | <0.0001 |  |
| 3 | Boy | 42.6 ± 0.2 (2) | 94.2 ± 7.0 | <0.0001 |  |
| 4 | Boy | 20.7 ± 4.6 (2) | 85.0 ± 2.5 | <0.0001 |  |
| 5 | Boy | 54.8 ± 6.2 (2) | 82.7 ± 1.9 | <0.0001 |  |
| 5 | Boy | 54.4 ± 2.4 (2) | 88.8 ± 0.5 | <0.0001 |  |
| 4 | Boy | 59.2 ± 1.8 (2) | 63.0 ± 3.0 | <0.0001 |  |
| 3 | Boy | 45.2 ± 0.6 (2) | 68.6 ± 1.1 | <0.0001 |  |
| 3 | Boy | 60.4 ± 0.9 (2) | 86.9 ± 4.6 | <0.0001 |  |
| 4 | Boy | 53.0 ± 3.5 (2) | 88.2 ± 3.5 | <0.0001 |  |
| 4 | Boy | 66.0 ± 2.6 (2) | 92.0 ± 2.6 | <0.0001 |  |
| 4 | Boy | 59.3 ± 4.6 (2) | 83.7 ± 3.1 | <0.0001 |  |
| 4 | Boy | 74.0 ± 2.6 (2) | 76.8 ± 3.7 | <0.0001 |  |
| 3 | Boy | 65.0 ± 1.6 (2) | 84.2 ± 2.2 | <0.0001 |  |
| 4 | Girl | 65.7 ± 0.8 (2) | 77.1 ± 1.7 | <0.0001 |  |
| 4 | Boy | 63.1 ± 1.9 (2) | 77.1 ± 1.7 | <0.0001 |  |
| Average | | 56.4 ± 2.8 | 79.9 ± 2.7 |  |  |
| 3 | Boy | 70.0 ± 3.9 (3) | 74.2 ± 1.2 | <0.0001 |  |
| 3 | Girl | 50.2 ± 3.3 (3) | 68.7 ± 1.1 | <0.0001 |  |
| 3 | Girl | 71.3 ± 2.9 (3) | 93.4 ± 1.4 | <0.0001 |  |
| 3 | Boy | 51.6 ± 1.6 (3) | 71.6 ± 1.5 | <0.0001 |  |
| 3 | Boy | 54.0 ± 1.2 (3) | 85.9 ± 3.1 | <0.0001 |  |
| 4 | Boy | 61.5 ± 3.6 (3) | 80.7 ± 7.3 | <0.0001 |  |
| Average | | 59.7 ± 2.8 | 78.9 ± 2.6 |  |  |

In our cohort, the average family income of our control group is higher. The reasons are discussed within the manuscript. However, there is no correlation between the family income difference and the urine FTIR spectra. Spectra of five children from the control group were selected and overlaid in Figure S1. Two of them are from families with the lowest income (#1 based on the numerical order provided in Table 2) and three are from those of the highest (#5). The spectra show absorbance differences in the region 3000-2600/cm; however, the degree of these differences among them is very small compared to that between the control group and the ASD+ group (Fig.S1-inlet). The positions of the five spectra in the inlet figure show no clear distinction that can be correlated with the family income difference. Therefore, the absorbance difference in the 3000-2600/cm region is attributed to reasons other than the family income differences.


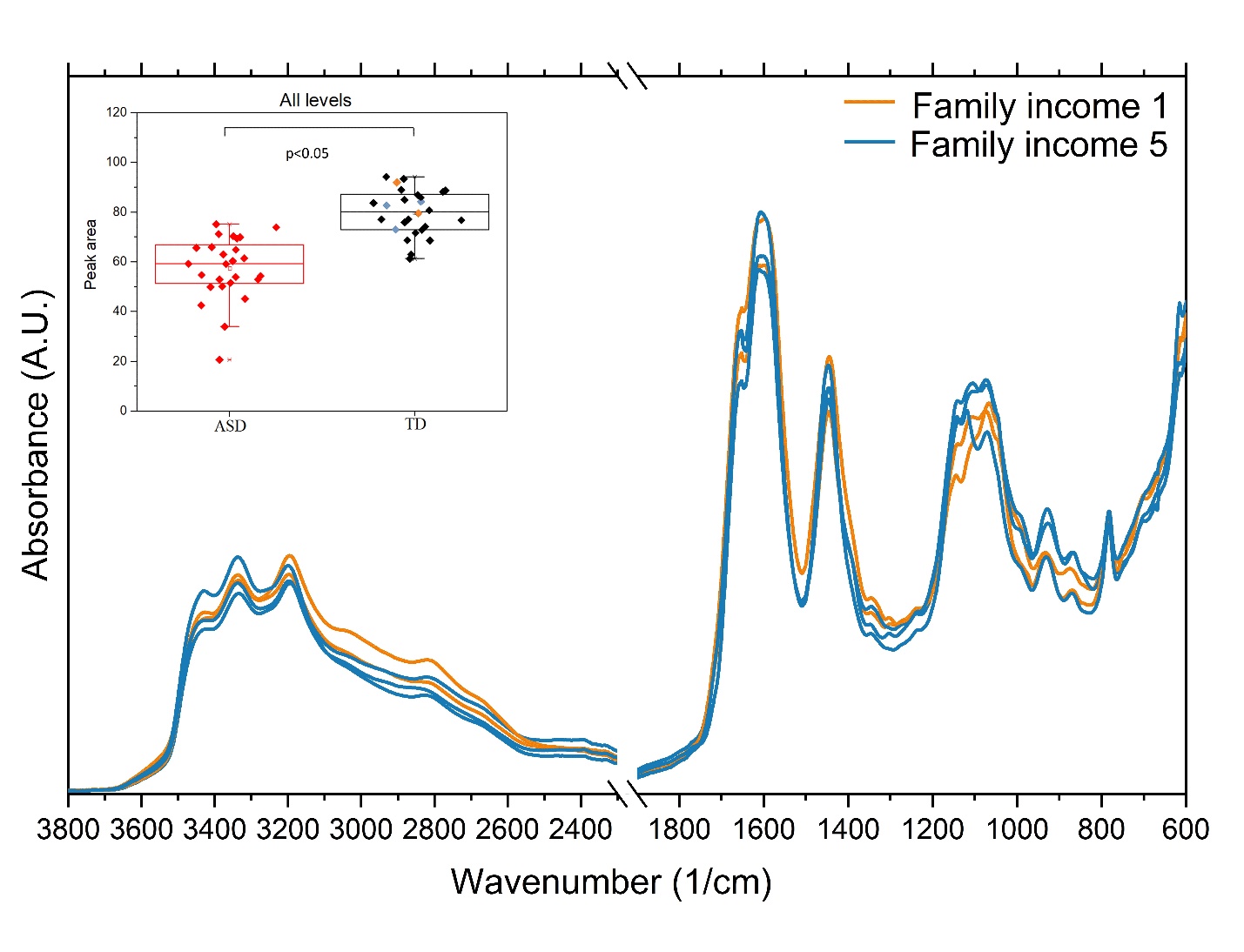


Figure S1. Spectra of five children from the control group are overlaid. Spectra are not pre-processed. Based on the numerals given in Table 2, two spectra (orange) represent family income 1 (the lowest) and three spectra (blue) represent family income 5 (the highest). The inlet figure shows a comparison of calculated area of the 3000-2600/cm region. The five spectra are shown with the same color coding in the inlet.
